# *Mycobacterium tuberculosis* Beijing Genotype Is Associated with HIV Infection in Mozambique

**DOI:** 10.1371/journal.pone.0071999

**Published:** 2013-08-07

**Authors:** Sofia O. Viegas, Adelina Machado, Ramona Groenheit, Solomon Ghebremichael, Alexandra Pennhag, Paula S. Gudo, Zaina Cuna, Egídio Langa, Paolo Miotto, Daniela M. Cirillo, Nalin Rastogi, Rob M. Warren, Paul D. van Helden, Tuija Koivula, Gunilla Källenius

**Affiliations:** 1 National Institute of Health, Ministry of Health, Maputo, Mozambique; 2 Faculty of Veterinary, Eduardo Mondlane University, Maputo, Mozambique; 3 Department of Clinical Science and Education, Södersjukhuset-Karolinska Institutet, Stockholm, Sweden; 4 Department of Preparedness, Swedish Institute for Communicable Disease Control, Solna, Sweden; 5 Tuberculosis National Control Program, Ministry of Health, Maputo, Mozambique; 6 Jhpiego Mozambique, an affiliate of Johns Hopkins University, Maputo, Mozambique; 7 Emerging Bacterial Pathogens Unit, San Raffaele Scientific Institute, Milan, Italy; 8 WHO Supranational TB Reference Laboratory, Tuberculosis & Mycobacteria Unit, Institut Pasteur de la Guadeloupe, Guadeloupe, France; 9 DST/NRF Centre of Excellence for Biomedical Tuberculosis Research/MRC Centre for Molecular and Cellular Biology, Division of Molecular Biology and Human Genetics, Faculty of Health Sciences, Stellenbosch University, Tygerberg, Western Cape, South Africa; Swiss Tropical and Public Health Institute, Switzerland

## Abstract

The Beijing genotype is a lineage of *Mycobacterium tuberculosis* that is distributed worldwide and responsible for large epidemics, associated with multidrug-resistance. However, its distribution in Africa is less understood due to the lack of data. Our aim was to investigate the prevalence and possible transmission of Beijing strains in Mozambique by a multivariate analysis of genotypic, geographic and demographic data. A total of 543 *M. tuberculosis* isolates from Mozambique were spoligotyped. Of these, 33 were of the Beijing lineage. The genetic relationship between the Beijing isolates were studied by identification of genomic deletions within some Regions of Difference (RD), Restriction Fragment Length Polymorphism (RFLP) and Mycobacterial Interspersed Repetivie Unit – variable number tandem repeat (MIRU-VNTR). Beijing strains from South Africa, representing different sublineages were included as reference strains. The association between Beijing genotype, Human Immunodeficiency Virus (HIV) serology and baseline demographic data was investigated. HIV positive serostatus was significantly (p=0.023) more common in patients with Beijing strains than in patients with non-Beijing strains in a multivariable analysis adjusted for age, sex and province (14 (10.9%) of the 129 HIV positive patients had Beijing strains while 6/141 (4.3%) of HIV negative patients had Beijing strains). The majority of Beijing strains were found in the Southern region of Mozambique, particularly in Maputo City (17%). Only one Beijing strain was drug resistant (multi-drug resistant). By combined use of RD and spoligotyping, three genetic sublineages could be tentatively identified where a distinct group of four isolates had deletion of RD150, a signature of the “sublineage 7” recently emerging in South Africa. The same group was very similar to South African “sublineage 7” by RFLP and MIRU-VNTR, suggesting that this sublineage could have been recently introduced in Mozambique from South Africa, in association with HIV infection.

## Background

Despite global efforts to combat tuberculosis (TB), the disease remains a major public health problem worldwide, especially in low resources countries such as Mozambique. Key factors for TB control are rapid detection, adequate therapy and infection control measures in place to prevent further transmission. Molecular typing methods have become powerful tools in TB epidemiology, to identify specific strains of *Mycobacterium tuberculosis* in order to monitor changes in microbial populations and to control outbreaks, to unveil hidden routes of transmission, and to survey the dissemination of old and emergent strains [1].

The Beijing genotype is a lineage of *M. tuberculosis* that has a worldwide distribution [2,3], and is highly endemic in certain geographic areas throughout Eastern and Southeast Asia [4,5] and it is also found to be predominant in South Africa and Russia [6,7]. The importance of the Beijing lineage is further highlighted by the fact that it was reported to be associated with an increased febrile response in patients during treatment [8], Human Immunodeficiency Virus (HIV) infection [9], multidrug-resistance (MDR) [2,4,7], enhanced virulence [1] and ability to evade Bacillus Calmette Guerin (BCG) protective immunity [10]. In South Africa, it was found that a specific Beijing sublineage, namely sublineage 7, was associated with increased transmissibility and/or pathogenicity [11].

We have previously reported [12] on the genotypic lineages of 445 *M. tuberculosis* isolates collected from the North and South regions of Mozambique. The Beijing family was found to be the fourth predominant lineage, and the Beijing Shared International Type (SIT) 1 was the third most frequent single spoligotype in Mozambique.

Here we investigate in depth the isolates belonging to the Beijing lineage, by extending the study to include isolates also from the Central Region of Mozambique. The Beijing strains are described in more detail by patients’ demographic data and by extended genotyping (Region of Difference (RD) analysis, Restriction Fragment Length Polymorphism (RFLP) and Mycobacterial Interspersed Repetivie Unit – Variable Number Tandem Repeat (MIRU-VNTR)) to compare their fingerprinting patterns with results of Beijing isolates from the neighboring South Africa. Our aim was to investigate the prevalence and possible transmission of Beijing strains in Mozambique.

## Materials and Methods

### Ethical considerations

Institutional permission to conduct the study was obtained from the National Bioethics Committee of the Ministry of Health in Maputo, Mozambique, reference number 148/CNBS/07. The patients were included in the resistance survey after understanding the study and having signed an informed consent. They were HIV tested after complete voluntary acceptance.

### Clinical isolates

A nationwide drug resistance survey was performed over one year (2007-8) by the National TB Control Program in 40 randomly selected diagnostic centers around the country. In the present study a total of 543 *M. tuberculosis* isolates from a bank of 1124 samples collected during the survey were studied based on viable organisms after re-culturing, 288 from the South region (95 from Maputo City, 92 from Maputo Province, 47 from Gaza and 54 from Inhambane), 91 from the Central Region (66 from Sofala, 17 from Manica and 8 from Tete) and 164 from the North Region (65 from Nampula, 76 from Cabó Delgado and 23 from Niassa). Of these 543 isolates, 445 strains have been previously reported [12].

Of the isolates studied, 536 were new cases (i.e. patients with pulmonary TB who had never been treated for TB or had been treated for less than 30 days) while 7 were cases previously treated (i.e. patients with pulmonary TB who were re-treatment cases and had a history of TB treatment for more than 30 days).

Basic demographic data was collected for each patient using a standard questionnaire. Patients were offered HIV-testing, and for those consenting HIV-testing was performed. The relationship between Beijing genotype, HIV serology, location and baseline demographic data was investigated.

### South African isolates

In order to compare with Mozambican Beijing strains, 13 previously characterised isolates from South Africa representing different Beijing sublineages were included in the study as reference strains and genotyped by IS*6110*-RFLP [11].

### Drug Susceptibility Testing

In order to evaluate the resistance pattern for isoniazid, rifampicin, streptomycin and ethambutol, the Resistance Ratio method was used [13].

### Spoligotyping

Deoxyribonucleic acid (DNA) was extracted using a standardized protocol [14]. Spoligotyping [15] was performed generally as described by Kamerbeek and colleagues using commercially available kit (Isogen Life Science B.V., Utrecht, The Netherlands). Spoligotyping results were analysed with the BioNumerics Software ver. 5.01 (Applied Maths, Kortrijk, Belgium).

### Region of Difference (RD) polymorphism

The identification of the genomic deletions RD105, RD142, RD150 and RD181 was done by PCR using primers previously described [16]. PCR conditions were 10 mM Tris–HCl (pH 8.8), 1.5 mM MgCl_2_, 50 mM KCl, 0.1% Triton X-100, 0.5 mM primers, 0.2 mM deoxynucleoside triphosphates, 1 U of Taq polymerase (Dynazyme) and 10 ng DNA per 50 ml of reaction mixture. PCR amplification was performed at the following conditions: 95^o^C for 15 min, followed by 35 cycles of 94^o^C for 1 min, 62^o^C for 1 min, and 72^o^C for 3 min. Ten-microlitre aliquots of PCR products were analyzed by 2% agarose gel electrophoresis.

### Insertion Sequence 6110 Restriction Fragment Length Polymorphism (IS*6110*-RFLP)

The isolates were cultured on Löwenstein-Jensen medium, DNA was extracted and RFLP typing was performed using the insertion sequence IS*6110* as a probe and *Pvu*II as the restriction enzyme [17]. Visual bands were analyzed using the BioNumerics software v 5.01 (Applied Maths, Kortrijk, Belgium). Strains with identical RFLP patterns (100% similarity) were judged to belong to a cluster. On the basis of the molecular sizes of the hybridizing fragments and the number of IS*6110* copies of each isolate, fingerprint patterns were compared by the un-weighted pair-group method of arithmetic averaging using the Jaccard coefficient. Dendrograms were constructed to show the degree of relatedness among strains according to a previously described algorithm [18] and similarity matrixes were generated to visualize the relatedness between the banding patterns of all isolates.

### Mycobacterial Interspersed Repetitive Unit – Variable Number Tandem Repeat (MIRU-VNTR) analysis

Standardized 24-loci MIRU-VNTR typing [19] was performed using the MIRU-VNTR typing kit (Genoscreen, Lille, France). The PCR-products were run with 1200 LIZ size standard (GeneScan, Applied Biosystems) on ABI3131xl sequencers. Sizing of the PCR-fragments and assignments of MIRU-VNTR alleles were done with the GeneMapper software version 4.1 (Applied Biosystems) according to the manufacturers’ instructions.

### HIV testing

HIV testing was performed according to the recommendations by the Ministry of Health, Mozambique at the clinical unit of enrolment. Two rapid HIV tests were used sequentially, Unigold Recombinant HIV (Trinity Biotech, Wicklow, Ireland) and Determine HIV-1/2 (Abbot, Tokyo, Japan). Samples were tested first with Determine and reported only when negative. Positive samples were confirmed with Unigold. All tests were done and interpreted according to the manufacturer’s instructions.

### Statistics

Univariate and multivariate logistic regression models were estimated for Beijing lineage as outcome and sex, age and HIV status and province (Maputo City or other) included as covariates. Interactions were tested for within the multivariable model but since no interactions were statistically significant they are not presented. The Hosmer-Lemeshaw goodness-of-fit test for the multivariate model was 0.452 and the fit of the model can therefore be considered as acceptable. Outliers were checked by means of the dfbetas and a possible outlier was detected but since the results did not change when the multivariable model was re-estimated excluding this observation all observations were included. The continuous variable age was deemed linear when assessed by means of the partial residuals. The largest variance inflation factor was 1.07 which indicates that there was no problem with multi-co-linearity.

The level of significance was set to 0.05 (two-sided) for all analyses. All analyses were performed in R v 2.9.2 (R Foundation for Statistical Computing, Vienna, Austria).

## Results

### Patients

In the present study, a total of 33 (6.1%) of 543 *M. tuberculosis* isolates were assigned as Beijing genotype by spoligotyping (31 isolates were from new cases and two were from previously treated patients).

The patients’ demographic data are summarized in Table 1. There was no significant association between Beijing genotype and age or gender (Supplementary table 1). A summary of the predominant lineages among the 543 *M. tuberculosis* isolates is presented in Supplementary Table 2.

**Table 1:** Patient demographic data.

		**Beijing (%)**	**Non-Beijing (%)**	**Total**
**Category**	**Total isolates**	33	510	543
**Gender**	Male	19 (57.6)	322 (63.1)	341
	Female	14 (42.4)	188 (36.9)	202
**TB case**	New	31 (93.9)	505 (99.0)	536
	Retreatment	2 (6.1)	5 (0.1)	7
**HIV sero-status**	Positive	14 (42.4)	115 (22.5)	129
	Negative	6 (18.2)	135 (26.5)	141
	Not tested for HIV	13 (39.4)	260 (50.1)	273

### HIV status in relation to Beijing genotype

Among all patients with Beijing strains, 20 (60.6%) were tested for HIV. The mean age of patients that were tested for HIV was 44.5 (SD 11.6) and the median age of those who did not consent to HIV testing was 39.9 years (SD 9.8) respectively.

Fourteen (10.9%) of the 129 HIV positive patients had Beijing strains while 6/141 (4.3%) of HIV negative patients had Beijing strains (Table 1). Thus HIV positive serostatus was significantly (p=0.049) more common in patients with Beijing strains than in patients with non-Beijing strains in a univariate analysis (Supplementary table 1). In a multivariate analysis (adjusted for age, sex and province) the correlation remained significant (p=0.023, Supplementary table 1).

### Geographic distribution of Beijing strains

**Figure 1:**
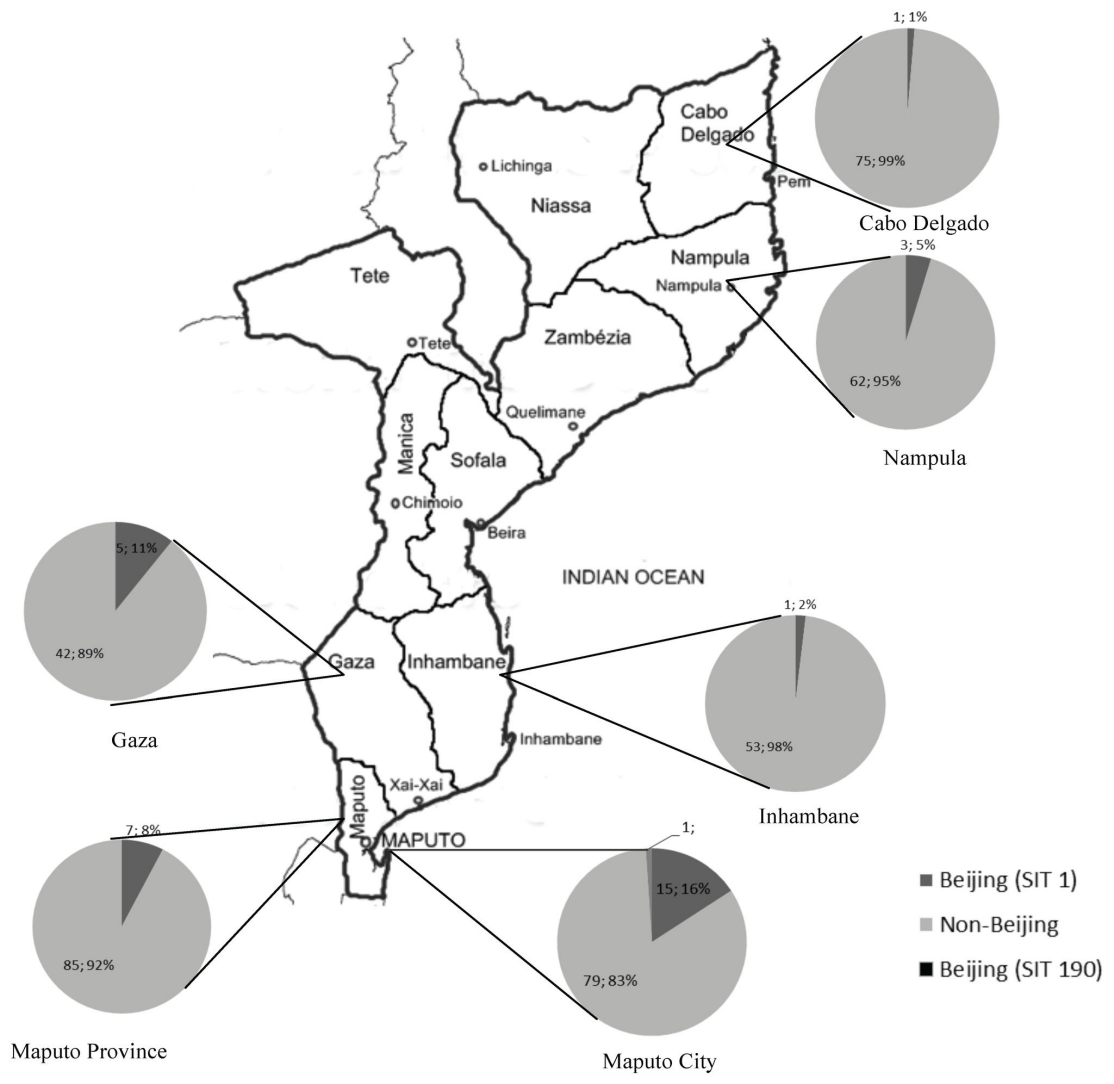
Distribution of Beijing genotype in Mozambique. Map of Mozambique showing the distribution of Beijing genotype in the country, prevalence among the total number of isolates per province. In the provinces of Sofala, Manica, Tete and Niassa non Beijing strain was found.

Figure 1 shows a map of the distribution of the Beijing strains in relation to non-Beijing strains among the different provinces of Mozambique. The majority of the Beijing strains were found in the Southern region (n=29) where the prevalence was 10.1% (29/288) while in the North the prevalence was 2.4%, (4/164). In the Central region, none of 91 isolates were of Beijing genotype.

In the Southern region we found that, although present in the four provinces, the Beijing lineage was most common in Maputo City 16/95 (16.8%), compared to the other provinces (p<0.001, Supplementary table 1). In a multivariate analysis the correlation remained significant between Beijing genotype isolates and Maputo City (p=0.004, Supplementary table 1).

The distribution of the fourteen HIV positive patients with Beijing genotype was as follow: 7 (50%) from Maputo City, 4 (28.6%) from Maputo Province, 2 (14.3%) from Gaza and 1 (7.1%) from Inhambane.

### Drug resistance

The results of the drug resistance survey provided by the National TB Control Program indicates that the MDR prevalence in Mozambique is 3.5% and 11.2% for new and previously treated cases respectively [20]. Only one (isolate 158, from a new case) of the 33 Beijing strains was drug resistant, being resistant to Rifampicin (RIF), Isoniazid (INH) and Streptomycin (STR) and susceptible to Ethambutol (EMB), i.e. by definition multidrug-resistant. The strain was from a 29 year old HIV positive male patient from Maputo Province.

### Molecular polymorphisms of *M. tuberculosis* Beijing genotype isolates

#### Spoligotyping and RD polymorphism

Using spoligotyping, Beijing genotype isolates were identified by the deletion of spacers 1–34, and the presence of at least three of the nine spacers 35–43 in the direct repeat locus of the *M. tuberculosis* genome [10,16,21]. Of the 33 strains that were defined by spoligotyping to be of the Beijing genotype, 32 had all the characteristic spacers 35-43, corresponding to the shared type SIT1 as defined in SITVIT2 (Table 2). One strain (isolate 35) in addition lacked spacer 40, corresponding to SIT190.

**Table 2:** Polymorphisms of *M. tuberculosis* Beijing genotype isolate.

		**Spoligotype description**		**Region of difference (RD)^a^**			
**Genotypic sublineage**	**Number of isolates (n=32^b^)**	**SIT^c^**	**Binary format^d^**	**105**	**142**	**150**	**181**
A	27	1	□□□□□□□□□□□□□□□□□□□□□□□□□□□□□□□□□□■■■■■■■■■	−	+	+	−
B	1	190	□□□□□□□□□□□□□□□□□□□□□□□□□□□□□□□□□□■■■■■□■■■	−	+	+	−
C	4	1	□□□□□□□□□□□□□□□□□□□□□□□□□□□□□□□□□□■■■■■■■■■	−	+	−	−

^a^ absence (−) or presence (+) of the specific genomic region

^b^ RD was not performed in one strain because there was no DNA

^c^ spoligotype international type, designations were assigned according to the definition in the SITVIT2 database.

^d^ The black and white boxes indicate the presence and absence, respectively, of the specific spacer at positions 1–43 in the DR locus.

Thirty two of the Beijing genotype strains (31 SIT1 isolates and the SIT190 isolate) were analysed for RD deletions. One isolate was not analysed because there was insufficient DNA. The majority of the isolates (n = 28) had the RD105 and RD181 deletions, while RD150 and RD142 were intact. Four strains lacked RD105, RD181 and RD150 while RD142 was intact (Table 2).

By a combined use of RD deletions and spoligotyping the 32 Beijing strains could be tentatively divided into three genetic sublineages, A, B and C (Table 2).

Sublineage A was the predominant sublineage, including 27 SIT1 isolates, with deletions of RD105 and RD181.

The sublineage B including one SIT190 isolate, had the same deletions of RD105 and RD181, but lacked spacer 40 by spoligotyping. The isolate was from a 19 year old woman, with unknown HIV status, from Maputo City.

The sublineage C, including four SIT1 isolates (isolate 46, 55, 327 and 1530), had deletions of RD105, 150 and 181. Deletion of RD150 represents a signature of the “sublineage 7” which is emerging in South Africa [11]. Of these four isolates, two (isolates 55 and 327) were from Maputo City (male, HIV positive patient and female, unknown HIV status), and one each from Gaza Province (female, HIV positive patient) and Cabó Delgado Province (male, unknown HIV status). The four patients were all young (20, 33, 20 and 19 years of age).

#### IS*6110*-RFLP

**Figure 2:**
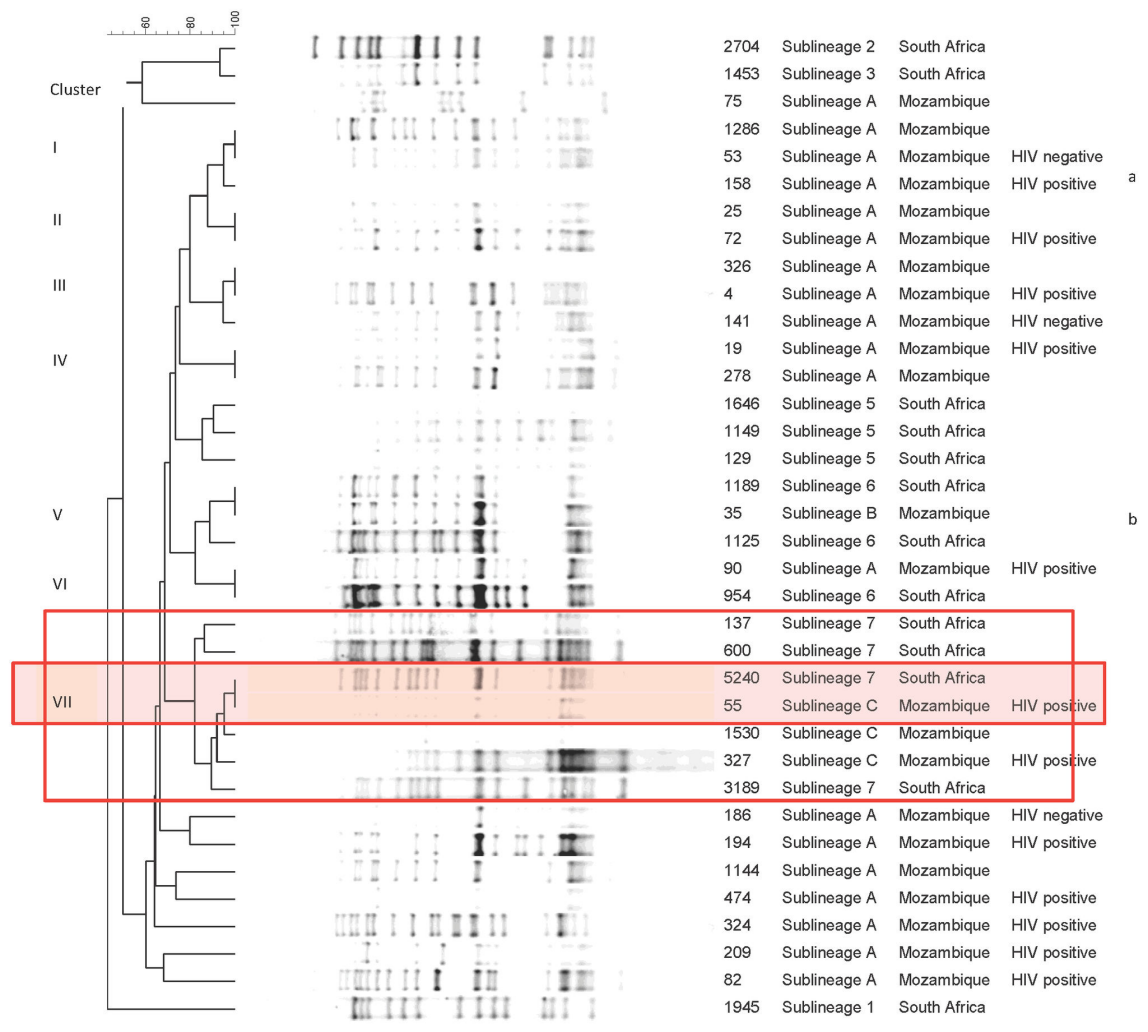
IS*6110* RFLP dendrogram of Beijing genotype strains from Mozambique and South Africa. The dendogram includes 36 *M*. *tuberculosis* Beijing genotype strains, 23 from Mozambique and 13 from South Africa. Red rectangle indicates sublineage 7 and sublineage C isolates from South Africa and Mozambique respectively; Highlighted area shows the clustered isolates from both sublineages. ^a^ Drug resistant isolate ^b^ SIT 190 isolate

RFLP was performed on 23 Beijing genotype isolates from Mozambique and 13 Beijing reference strains from South Africa and were compared for similarities. Ten isolates from Mozambique were not analysed due to insufficient DNA. Of the Mozambican Beijing strains, there were four clusters with two isolates each as defined by identical IS*6110* RFLP patterns (cluster I, II, III and IV). The remaining 15 isolates from Mozambique had unique IS*6110* RFLP patterns yielding a total of 19 different patterns (Figure 2). When compared to South African isolates, 3 additional clusters (cluster V, VI and VII) were obtained, each cluster containing one isolate per country.

Cluster V contained isolate 35, the Mozambican SIT190 isolate. Cluster VII contained isolate 55 (from a HIV positive patient), one of the sublineage C isolates, which clustered with one of the South African “sublineage 7” isolates (Figure 2, marked in red). Isolates 1530 and 327 (from a HIV positive patient), of sublineage C, were also very similar to the South African “sublineage 7” isolates in terms of RFLP pattern (Figure 2). RFLP was not performed on isolate 46 of sublineage C.

#### MIRU-VNTR

**Figure 3:**
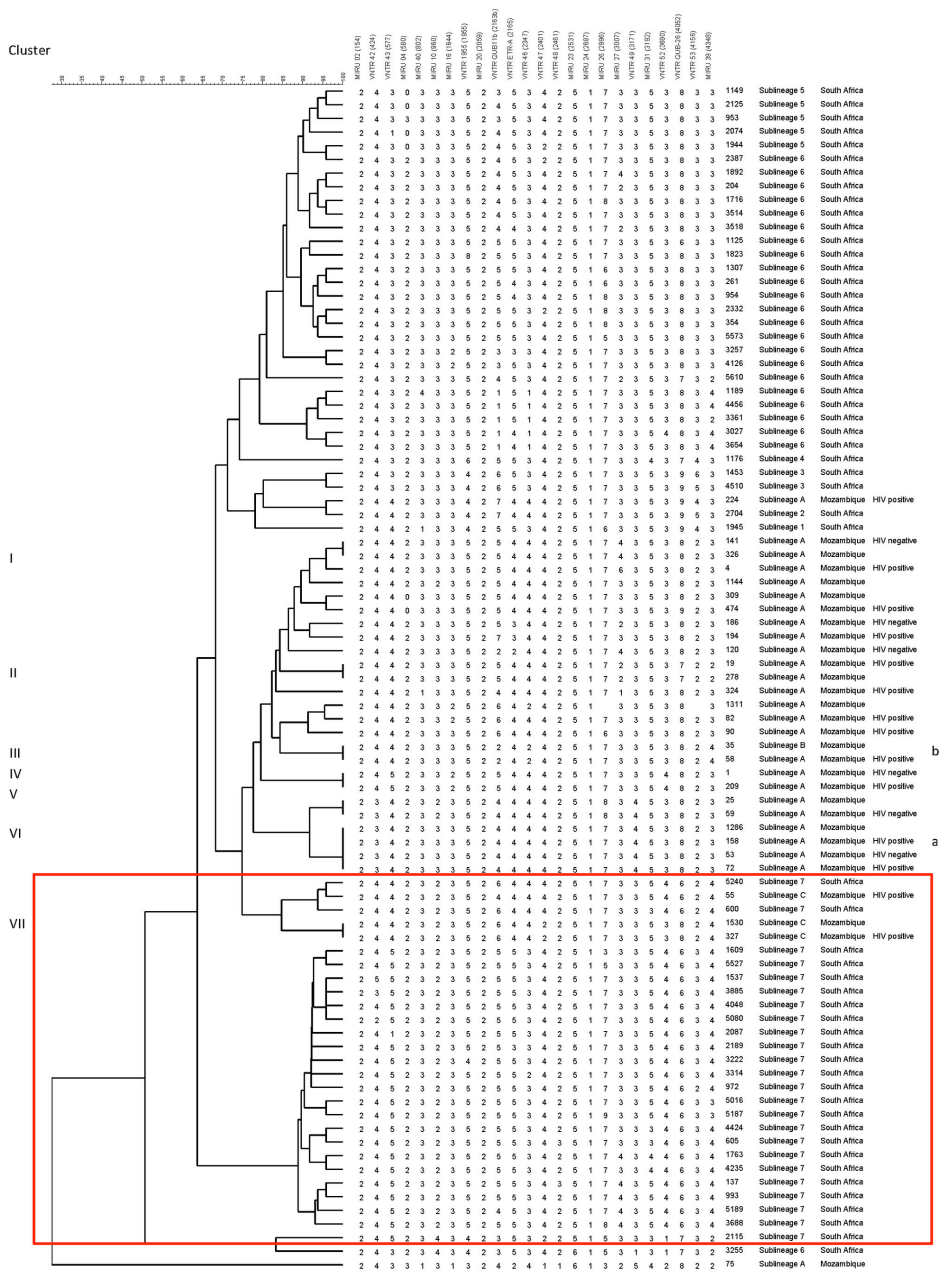
24 loci MIRU-VNTR dendrogram of Beijing genotype strains from Mozambique and South Africa. The dendrogram includes 87 *M*. *tuberculosis* Beijing genotype strains, 30 from Mozambique and 57 from South Africa. Red rectangle indicates sublineage 7 and sublineage C isolates from South Africa and Mozambique respectively. ^a^ Drug resistant isolate ^b^ SIT 190 isolate

MIRU-VNTR analysis was done on 30 Beijing strains from Mozambique and the obtained results were compared with 54 isolates from South Africa previously described [22], each one with one unique pattern. The isolates from Mozambique formed seven clusters (2 or 4 isolates per cluster) (Figure 3). Cluster II, III, IV and VII had one HIV positive patient and cluster VI had two HIV positive patients.

Although the Mozambican sublineage C isolates analyzed (55, 327 and 1530) did not cluster by 24 loci MIRU-VNTR to any of the South African “sublineage 7” isolates, they had similar patterns (Figure 3, marked with a red rectangle). MIRU-VNTR was not performed on three isolates from Mozambique, including isolate 46 of sublineage C.

## Discussion

In this study of *M. tuberculosis* strains, collected during a one year drug resistance survey in Mozambique, 33 (6%) of 543 strains were of the Beijing genotype. The fact that a significant number of patients with Beijing strains were HIV positive is worrying. Beijing strains have recently been reported to be associated with HIV positive serostatus also in South Africa [9], a connection which is further supported here. This association can be due to a combination of increased virulence of the strains and an increased susceptibility of HIV infected patients to these strains. Only one of the Beijing isolates was drug resistant, although Beijing strains have been associated with multidrug resistant TB [7].

The patients with Beijing strains were significantly over-represented in Maputo City, and the low prevalence in the Central and Northern parts of the country suggests that Maputo City and its surroundings is the likely origin.

Analysis of large sequence polymorphisms has shown that the Beijing lineage has evolved into distinct branches defined by specific RD deletions. The large deletion of RD105 is considered to be a marker for Beijing strains [11,16,23], although deletion of the RD105 was recently found also in ancestral strains with non-Beijing spoligoprofiles [24]. All Beijing genotype isolates (defined by spoligotyping) in this study had the RD105 deletion. Additional deletions of RD142, RD150 and RD181 may further divide this family into different sublineages [16]. No isolate showed deletion of the RD142 region, in accordance with the low frequency of this deletion event reported elsewhere [13,18]. Interestingly, 4 out of 32 isolates (sublineage C) lacked RD150, a relatively rare deletion, present in a recently evolved sublineage, “sublineage 7”, in South Africa, reported to be associated with increased transmissibility and/or pathogenicity [11]. Moreover the sublineage C isolates were very similar to South African sublineage 7 by 24-loci MIRU-VNTR and RFLP.

Our findings on the low clonality of the strains by MIRU-VNTR and RFLP demonstrate that the population structure of the Beijing genotype in Mozambique consists of more than one sublineage, indicating that these strains were introduced to the country on separate occasions.

Mozambique and South Africa are neighbouring countries and have a history of cross boarder migration. The fact that four of the Beijing genotype isolates had the RD150 deletion, were clustered or were similar by RFLP with South African “sublineage 7” isolates [11], and also by MIRU-VNTR were close to the South African “sublineage 7” isolates, and considering the high endemicity of this sublineage in South Africa and the low prevalence in Mozambique (4/33) suggests that this sublineage could have been recently introduced in Mozambique from South Africa.

An association with positive HIV serostatus has been reported for other *M. tuberculosis* lineages. In Malawi, lineage 1 (“Indo-Oceanic”) strains were more common in those with HIV infection, even after adjusting for age and sex [25]. In Nigeria, LAM10-CAM, with phylogeographical specificity for Cameroon and neighboring countries in West Africa was significantly more common in HIV-positive TB patients [26].

When we analyzed the association between HIV infection and the other most prevalent lineages in the country, an association with HIV was found for the LAM lineage, but for the EAI and T lineages no association with HIV was observed (data not shown), this finding warrants further investigation.

There are certain limitations of the present study. The study is based on a sample of isolates from a drug resistance survey, and may not reflect the true population structure. The low number of viable specimens (543 *M. tuberculosis* isolates from a bank of 1124), and a low number of Beijing genotype strains, and the fact that HIV status was not determined for all patients and that not all isolates were genotyped by RFLP and MIRU-VNTR are further limitations of the study. For this reason further longitudinal studies are indicated, both to test the hypothesis that the Beijing genotype is emerging in Mozambique, and to further investigate the potential role of HIV infection in this setting.

We recommend in the near future introduction of molecular genetic methods at reference level in Mozambique, particularly for migrant patients, with emphasis on mine workers from South Africa in order to control the transmission between countries, as well as for HIV positive individuals for monitoring possible epidemics related to opportunistic strains and drug resistance.

## Supporting Information

Table S1 **Logistic regression analysis of demographic data.**
(DOCX)

Table S2 **Summary of the predominant *M. tuberculosis* lineages.**
(DOCX)
