# Supplementary material for: Mycobacterium tuberculosis Beijing Genotype Is Associated with HIV Infection in Mozambique
Source: PLoS One. 2013 Aug 7;8(8):e71999. doi: 10.1371/journal.pone.0071999 (PMC3737140; doi:10.1371/journal.pone.0071999)
Supplement: Table S1 — Logistic regression analysis of demographic data. (DOCX) [file pone.0071999.s001.docx]

|  | Univariable | | | | Multivariable | | | |
| --- | --- | --- | --- | --- | --- | --- | --- | --- |
|  | n | OR | 95%CI | p- value | n | OR | 95%CI | p- value |
| Age | 33/519 | 0.96 | 0.93-0.99 | 0.015 | 20/257 | 0.97 | 0.93-1.02 | 0.256 |
| Sex F vs M | 33/543 | 1.26 | 0.62-2.58 | 0.523 |  | 1.03 | 0.39-2.73 | 0.957 |
| Maputo City vs Other | 33/543 | 5.13 | 2.49-10.59 | <0.001 |  | 4.23 | 1.61-11.17 | 0.004 |
| HIV status (Positive vs Negative) | 20/268 | 2.70 | 1.00-7.25 | 0.049 |  | 3.35 | 1.19-9.50 | 0.023 |
