# Supplementary material for: Mycobacterium tuberculosis Beijing Genotype Is Associated with HIV Infection in Mozambique
Source: PLoS One. 2013 Aug 7;8(8):e71999. doi: 10.1371/journal.pone.0071999 (PMC3737140; doi:10.1371/journal.pone.0071999)
Supplement: Table S2 — Summary of the predominant M. tuberculosis lineages. (DOCX) [file pone.0071999.s002.docx]

| ***M. tuberculosis* lineage** | **Number of isolates** | **Percentage in relation to all isolates** |
| --- | --- | --- |
| Beijing | 33 | 6,1% |
| Latin American Mediterranean | 182 | 33,5% |
| East African Indian | 173 | 31,9% |
| T | 58 | 10,7% |
| Other | 97 | 17,9% |
| Total | 543 | 100,00% |
